# An Assessment of the Predictive Performance of Current Machine Learning–Based Breast Cancer Risk Prediction Models: Systematic Review

**DOI:** 10.2196/35750

**Published:** 2022-12-29

**Authors:** Ying Gao, Shu Li, Yujing Jin, Lengxiao Zhou, Shaomei Sun, Xiaoqian Xu, Shuqian Li, Hongxi Yang, Qing Zhang, Yaogang Wang

**Affiliations:** 1 Health Management Center, Tianjin Medical University General Hospital Tianjin China; 2 School of Management, Tianjin University of Traditional Chinese Medicine Tianjin China; 3 Department of Bioinformatics, School of Basic Medical Sciences, Tianjin Medical University Tianjin China; 4 School of Public Health, Tianjin Medical University Tianjin China

**Keywords:** breast cancer, machine learning, risk prediction, cancer, oncology, systemic review, review, meta-analysis, cancer research, risk model

## Abstract

**Background:**

Several studies have explored the predictive performance of machine learning–based breast cancer risk prediction models and have shown controversial conclusions. Thus, the performance of the current machine learning–based breast cancer risk prediction models and their benefits and weakness need to be evaluated for the future development of feasible and efficient risk prediction models.

**Objective:**

The aim of this review was to assess the performance and the clinical feasibility of the currently available machine learning–based breast cancer risk prediction models.

**Methods:**

We searched for papers published until June 9, 2021, on machine learning–based breast cancer risk prediction models in PubMed, Embase, and Web of Science. Studies describing the development or validation models for predicting future breast cancer risk were included. The Prediction Model Risk of Bias Assessment Tool (PROBAST) was used to assess the risk of bias and the clinical applicability of the included studies. The pooled area under the curve (AUC) was calculated using the DerSimonian and Laird random-effects model.

**Results:**

A total of 8 studies with 10 data sets were included. Neural network was the most common machine learning method for the development of breast cancer risk prediction models. The pooled AUC of the machine learning–based optimal risk prediction model reported in each study was 0.73 (95% CI 0.66-0.80; approximate 95% prediction interval 0.56-0.96), with a high level of heterogeneity between studies (*Q*=576.07, *I*^2^=98.44%; *P*<.001). The results of head-to-head comparison of the performance difference between the 2 types of models trained by the same data set showed that machine learning models had a slightly higher advantage than traditional risk factor–based models in predicting future breast cancer risk. The pooled AUC of the neural network–based risk prediction model was higher than that of the nonneural network–based optimal risk prediction model (0.71 vs 0.68, respectively). Subgroup analysis showed that the incorporation of imaging features in risk models resulted in a higher pooled AUC than the nonincorporation of imaging features in risk models (0.73 vs 0.61; *P*_heterogeneity_=.001, respectively). The PROBAST analysis indicated that many machine learning models had high risk of bias and poorly reported calibration analysis.

**Conclusions:**

Our review shows that the current machine learning–based breast cancer risk prediction models have some technical pitfalls and that their clinical feasibility and reliability are unsatisfactory.

## Introduction

Of all the cancers worldwide among women, breast cancer shows the highest incidence and mortality [1]. Early access to effective diagnostic and treatment services after breast cancer screening could have reduced breast cancer mortality by 25%-40% over the last several decades [2,3]. The development and implementation of risk-based breast cancer control and prevention strategies can have great potential benefits and important public health implications. Moreover, risk-based breast cancer control and prevention strategy is more effective and efficient than conventional screening based on model evaluation [4,5]. A prerequisite for the implementation of personalized risk-adapted screening intervals is accurate breast cancer risk assessment [6]. Models with high sensitivity and specificity can enable screening to target more elaborate efforts for high-risk groups while minimizing overtreatment for the rest. Currently, the US breast cancer screening guidelines use breast cancer risk assessments to inform the clinical course, thereby targeting the high-risk population by earlier detection and lesser screening harms (eg, false-positive results, overdiagnosis, overtreatment, increased patient anxiety) [7]. Nevertheless, there is no standardized approach for office-based breast cancer risk assessment worldwide.

Traditional risk factor–based models such as Gail, BRCAPRO, Breast Cancer Surveillance Consortium, Claus, and Tyrer-Cuzick models have been well-validated and used commonly in clinical practice, but these models developed by logistic regression or Cox regression or those presented as risk scoring systems have low discrimination accuracy with the area under the receiver operating characteristic curve (AUC) between 0.53 and 0.64 [8-12] and these models show bias when applied to minority populations, accompanied by great variance in terms of the patients included, methods of development, predictors, outcomes, and presentations [13-15]. Other risk prediction models that incorporated genetic risk factors were also only best suited for specific clinical scenarios and may have limited applicability in certain types of patients [16]. Recently, with the cross research between artificial intelligence and medicine, the development and validation of breast cancer risk prediction models based on machine learning algorithms have been the current research focus. Machine learning algorithms provide an alternative approach to standard prediction modelling, which may address the current limitations and improve the prediction accuracy of breast cancer susceptibility [17,18]. Mammography is the most commonly used method for breast cancer screening or early detection. Machine learning artificial intelligence models suggest that mammographic images contain risk indicators and germline genetic data that can be used to improve and strengthen the existing risk prediction models [19]. Some studies claim that machine learning–based breast cancer risk prediction models are better than regression method–based models [7,20], but 1 study reported the opposite result [21]. These controversial conclusions prompted us to review the performance and the weaknesses of machine learning–based breast cancer risk prediction models. Therefore, this systematic review and meta-analysis aims to assess the performance and clinical feasibility of the currently available machine learning–based breast cancer risk prediction models.

## Methods

### Study Protocol

This systematic review and meta-analysis was performed according to the PRISMA (Preferred Reporting Items for Systematic Reviews and Meta-Analysis) statement [22], the Checklist for Critical Appraisal and Data Extraction for Systematic Reviews of Prediction Modeling Studies, and the prediction model performance guidelines [23,24].

### Literature Search Strategy

Papers on machine learning–based breast cancer risk prediction models were searched in PubMed, Embase, and Web of Science by using the terms “machine learning OR deep learning” AND “mammary OR breast cancer OR carcinoma OR tumor OR neoplasm” AND “risk assessment OR risk prediction” published until June 9, 2021, and limited to papers published in English. The complete search strategy is detailed in Multimedia Appendix 1. Reviews in this field and references of the original papers were also manually checked to identify whether there were any missed studies.

### Inclusion and Exclusion Criteria

Studies describing development or validation models for predicting future breast cancer risk were included in our study. The inclusion criteria were as follows: (1) breast cancer risk prediction model developed using a machine learning algorithm, (2) mean follow-up period for cohort studies should be longer than 1 year, and (3) future breast cancer risk is the assessment result. The exclusion criteria were as follows: (1) review or conference or editorial or only published abstracts, (2) the original full text not available or incomplete information, and (3) studies with no AUC or C-statistic and its 95% CI. When papers included the same population, studies with larger sample size or longer follow-up periods were finally included.

### Data Extraction and Study Quality

Two researchers independently collected data on the first author, publication year, geographic region, study design, study population, sample size, study period, age of participants, time point for breast cancer risk prediction, name of the risk prediction model, number of participants and cancer cases in test data set, input risk factors, development and verification methods, and AUC with its 95% CI. The Prediction Model Risk of Bias Assessment Tool (PROBAST) was used to assess the risk of bias (ROB) and the clinical applicability of the included studies [25,26]. Any discrepancies were resolved by consensus or were consulted with the corresponding author.

### Statistical Analyses

The discrimination value was assessed by AUC, which measures the machining learning risk prediction model ability to distinguish the women who will and will not develop breast cancer. An AUC of 0.5 was considered as no discrimination, whereas 1.0 indicated perfect discrimination. We calculated the pooled AUC of the risk models by using DerSimonian and Laird’s random-effects model [27]. A head-to-head performance comparison of the studies that developed machine learning models and those that developed traditional risk factor–based models can help us understand the performance gain of utilizing machine learning methods in the same experimental setting. The *Q* test and *I*^2^ value were employed to evaluate the heterogeneity among the studies. High values in both tests (*I*^2^>40%, a significant *Q* test value with *P*<.05) showed high levels of inconsistency and heterogeneity. We also calculated an approximate 95% prediction interval (PI) to depict the extent of between-study heterogeneity [28]. Sensitivity analysis was performed to assess the influence of each study on the pooled effects by omitting each study. The visualized asymmetry of the funnel plot and Egger regression test were assessed for the publication bias. Pooled effects were also adjusted using the Duval and Tweedie trim-and-fill method [29,30]. All statistical meta-analyses of the predictive performance were performed using the MedCalc statistical software version 20 (MedCalc Ltd).

## Results

### Eligible Papers and Study Characteristics

**Figure 1:**
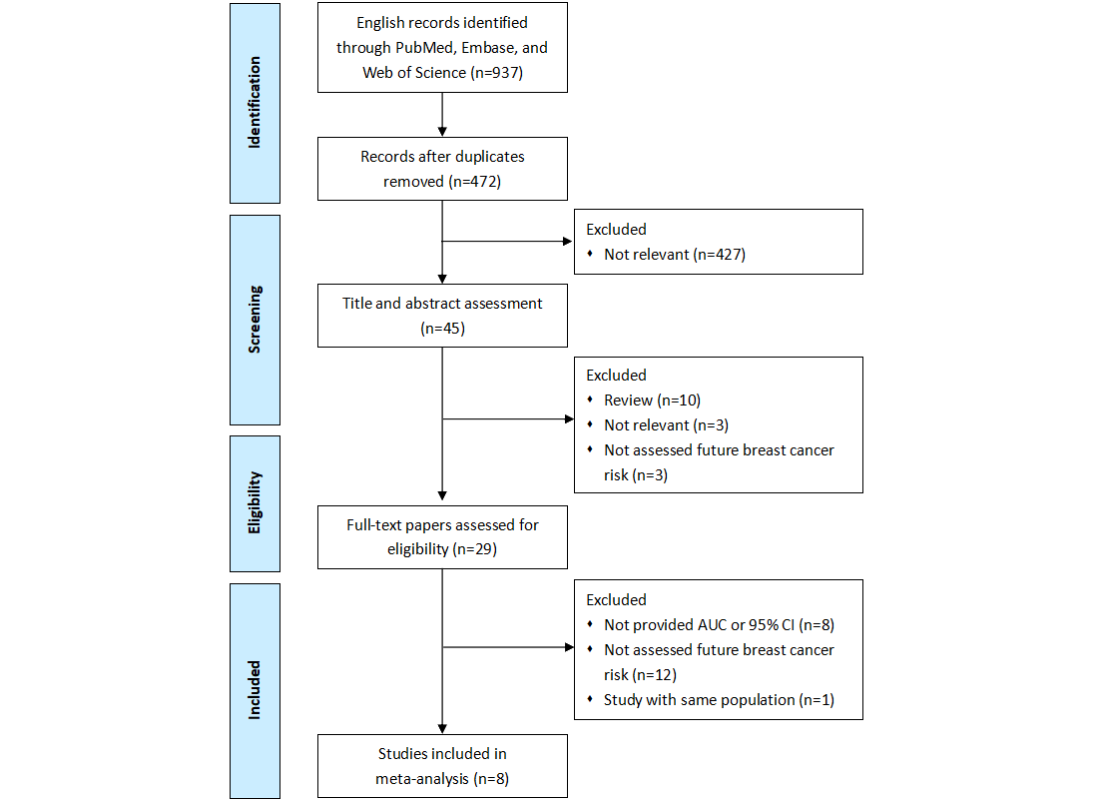
Flowchart of the study selection in this systematic review. AUC: area under the curve.

A total of 937 papers were identified, and 8 studies with 10 data sets met our inclusion criteria and they were finally included in the meta-analysis (Figure 1) [7,19-21,31-34]. The primary characteristics of the included studies are summarized in Table 1. Totally, 218,100 patients were included in this review. Most of these patients were from America and Europe; only 1 data set’s participants were from Taiwan, China. Six studies [7,20,21,32-34] predicted short-term (≤5 year) breast cancer risk, while 2 studies [19,31] predicted long-term (future or lifetime) risk. The characteristics and performance of the machine learning–based breast cancer risk prediction models are summarized in Table 2. Most of the machine learning prediction models were development models; only 1 study [7] used 3 different ethnic groups for external validation. Neural network was the most common machine learning method for the development of breast cancer risk prediction models. Only 1 neural network–based model incorporated genetic risk factors [7] and 6 neural network–based models incorporated imaging features [7,20,31,32].

**Table 1:** Characteristics of the included studies on the machine learning–based breast cancer risk prediction models.

Study ID	Study design	Study population, geographic location	Sample size	Age (years)	Study period	Breast cancer risk	Participants in test data set (n)	Cancers in test data set (n)
Yala et al [7], 2021	Retrospective study	Massachusetts General Hospital, USA	70,972	40-80	2009-2016	5 years	7005	588
Yala et al [7], 2021	Retrospective study	Cohort of Screen-Aged Women, Karolinska University Hospital, Sweden	7353	40-74	2008-2016	5 years	7353	1413
Yala et al [7], 2021	Retrospective study	Chang Gung Menoral Hospital, Taiwan	13,356	40-70	2010-2011	5 years	13,356	244
Ming et al [19], 2020	Retrospective study	Oncogenetic Unit, Geneva University Hospital, Sweden	45,110	20-80	1998-2017	Lifetime	36,146	4911
Portnoi et al [20], 2019	Retrospective study	A large tertiary academic medical center, Massachusetts General Hospital, USA	1183	40-80	2011-2013	5 years	1164	96
Stark et al [21], 2019	Prospective study	Prostate, Lung, Colorectal, and Ovarian Cancer Screening Trial data set, USA	64,739	50-78	1993-2001	5 years	12,948	269
Dembrower et al [31], 2020	Retrospective study	Cohort of Screen-Aged Women, Karolinska University Hospital, Sweden	14,034	40-74	2008-2015	Future	2283	278
Arefan et al [32], 2020	Retrospective case-control study cohort	Health Insurance Portability and Accountability Act, USA	226	41-89	2013	Short-term	226	113
Tan et al [33], 2013	Retrospective study	University of Oklahoma Medical Center, USA	994	—^a^	2006	12-36 months	994	283
Saha et al [34], 2019	Retrospective study	Duke University School of Medicine, USA	133	27-76	2004-2013	2 years	133	46

^a^Not available.

**Table 2:** Characteristics and performance of the machine learning–based breast cancer risk prediction models.

Study ID, model name			Statistical method		Development/validation model		Model input parameters		Incorporation of imaging features		AUC^a^ (95% CI)
**Yala et al [7], 2021**											
	Tyrer-Cuzick model^b^	Logistic regression		—^c^		Age, weight, height, menarche age, given birth, menopause status, hormone replacement therapy usage, *BRCA* gene, ovarian cancer, breast biopsy, family history, hormonal factors		No		0.62 (0.59-0.66)	
	Radiolosit BI-RADS^d^ model^e^	Logistic regression		Development model		Mammographic features		Yes		0.62 (0.60-0.65)	
	Image- and heatmaps model	Convolutional neural network		Development model		—		Yes		0.64 (0.60-0.68)	
	Imaged-only deep learning model	Convolutional neural network		Development model		Mammographic features		Yes		0.73 (0.70-0.77)	
	Hybrid deep learning model	Convolutional neural network		Development model		Age, weight, height, menarche age, given birth, menopause status, hormone replacement therapy usage, *BRCA* gene, ovarian cancer, breast biopsy, family history, hormonal factors		Yes		0.72 (0.69-0.76)	
	Mirai without risk factors model^f^	Convolutional neural network		Development model		Mammographic features		Yes		0.76 (0.73-0.79)	
	Mirai with risk factors model	Convolutional neural network		Development model		Age, weight, height, menarche age, given birth, menopause status, hormone replacement therapy usage, *BRCA* gene, ovarian cancer, breast biopsy, family history, hormonal factors		Yes		0.76 (0.73-0.80)	
**Yala et al [7], 2021**											
	Imaged-only deep learning model	Convolutional neural network		Validation model		Mammographic features		Yes		0.71 (0.69-0.73)	
	Mirai without risk factors model^f^	Convolutional neural network		Validation model		Mammographic features		Yes		0.78 (0.76-0.80)	
**Yala et al [7], 2021**											
	Imaged-only deep learning model	Convolutional neural network		Validation model		Mammographic features		Yes		0.70 (0.66-0.73)	
	Mirai without risk factors model^f^	Convolutional neural network		Validation model		Mammographic features		Yes		0.79 (0.75-0.82)	
**Ming et al [19], 2020**											
	BOADICEA^g^ model	Logistic regression		—		Family pedigree, age, age at menarche, age at first live birth, parity, age at menopause, Ashkenazi Jewish ancestry, ovarian, prostate, pancreatic, contralateral, and lung/bronchus cancer diagnosis and age of onset, estrogen receptor status, progesterone receptor status, *HER2* status, and *BRCA/BRCA2* germline pathogenic variant		No		0.639^h^	
	Machine learning-Markov Chain Monte Carlo generalized linear mixed model	Markov Chain Monte Carlo		Development model		Family pedigree, age, age at menarche, age at first live birth, parity, age at menopause, Ashkenazi Jewish ancestry, ovarian, prostate, pancreatic, contralateral, and lung/bronchus cancer diagnosis and age of onset, estrogen receptor status, progesterone receptor status, *HER2* status, and *BRCA/BRCA2* germline pathogenic variant		No		0.851 (0.847-0.856)	
	Machine learning-adaptive boosting model^e,f^	Adaptive boosting		Development model		Family pedigree, age, age at menarche, age at first live birth, parity, age at menopause, Ashkenazi Jewish ancestry, ovarian, prostate, pancreatic, contralateral, and lung/bronchus cancer diagnosis and age of onset, estrogen receptor status, progesterone receptor status, *HER2* status, and *BRCA/BRCA2* germline pathogenic variant		No		0.889 (0.875-0.903)	
	Machine learning-random forest model	Random forest		Development model		Family pedigree, age, age at menarche, age at first live birth, parity, age at menopause, Ashkenazi Jewish ancestry, ovarian, prostate, pancreatic, contralateral, and lung/bronchus cancer diagnosis and age of onset, estrogen receptor status, progesterone receptor status, *HER2* status, and *BRCA/BRCA2* germline pathogenic variant		No		0.843 (0.838-0.849)	
**Portnoi et al [20], 2019**											
	Traditional risk factors logistic regression model^e^	Logistic regression		Development model		Age, weight, height, breast density, age at menarche, age at first live birth, menopause, hormone replacement therapy usage, had gene mutation, had ovarian cancer, had breast biopsy, number of first-degree relatives who have had breast cancer, race/ethnicity, history of breast cancer, and background parenchymal enhancement on magnetic resonance images		No		0.558 (0.492-0.624)	
	Magnetic resonance image-deep convolutional neural network model^f^	Convolutional neural network		Development model		Full-resolution magnetic resonance images		Yes		0.638 (0.577-0.699)	
	Tyrer-Cuzick model^b^	Logistic regression		—		Age, weight, height, breast density, age at menarche, age at first live birth, menopause, hormone replacement therapy usage, had gene mutation, had ovarian cancer, had breast biopsy, number of first-degree relatives who have had breast cancer, and race/ethnicity, and history of breast cancer		No		0.493 (0.353-0.633)	
**Stark et al [21], 2019**											
	Feed-forward artificial neural network model	Artificial neural network		Development model		Age, age at menarche, age at first live birth, number of first-degree relatives who have had breast cancer, race/ethnicity, age at menopause, an indicator of current hormone usage, number of years of hormone usage, BMI, pack years of cigarettes smoked, years of birth control usage, number of liver births, an indicator of personal prior history of cancer		No		0.608 (0.574-0.643)	
	Logistic regression model^e,f^	Logistic regression		Development model		Age, age at menarche, age at first live birth, number of first-degree relatives who have had breast cancer, and race/ethnicity, age at menopause, an indicator of current hormone usage, number of years of hormone usage, BMI, pack years of cigarettes smoked, years of birth control usage, number of liver births, an indicator of personal prior history of cancer		No		0.613 (0.579-0.647)	
	Gaussian naive Bayes model	Gaussian naive Bayes		Development model		Age, age at menarche, age at first live birth, number of first-degree relatives who have had breast cancer, and race/ethnicity, age at menopause, an indicator of current hormone usage, number of years of hormone usage, BMI, pack years of cigarettes smoked, years of birth control usage, number of liver births, an indicator of personal prior history of cancer		No		0.589 (0.555-0.623)	
	Decision tree model	Decision tree		Development model		Age, age at menarche, age at first live birth, number of first-degree relatives who have had breast cancer, and race/ethnicity, age at menopause, an indicator of current hormone usage, number of years of hormone usage, BMI, pack years of cigarettes smoked, years of birth control usage, number of liver births, an indicator of personal prior history of cancer		No		0.508 (0.496-0.521)	
	Linear discriminant analysis model	Linear discriminant analysis		Development model		Age, age at menarche, age at first live birth, number of first-degree relatives who have had breast cancer, and race/ethnicity, age at menopause, an indicator of current hormone usage, number of years of hormone usage, BMI, pack years of cigarettes smoked, years of birth control usage, number of liver births, an indicator of personal prior history of cancer		No		0.613 (0.579-0.646)	
	Support vector machine model	Support vector machine		Development model		Age, age at menarche, age at first live birth, number of first-degree relatives who have had breast cancer, and race/ethnicity, age at menopause, an indicator of current hormone usage, number of years of hormone usage, BMI, pack years of cigarettes smoked, years of birth control usage, number of liver births, an indicator of personal prior history of cancer		No		0.518 (0.484-0.551)	
	Breast Cancer Risk Prediction Tool model^b^	Logistic regression		—		Age, age at menarche, age at first live birth, number of first-degree relatives who have had breast cancer, and race/ethnicity, age at menopause, an indicator of current hormone usage, number of years of hormone usage, BMI, pack years of cigarettes smoked, years of birth control usage, number of liver births, an indicator of personal prior history of cancer		No		0.563 (0.528-0.597)	
**Dembrower et al [31], 2020**											
	Deep learning risk score model	Deep neural network		Development model		Mammographic images, the age at image acquisition, exposure, tube current, breast thickness, and compression force		Yes		0.65 (0.63-0.66)	
	Dense area model^b,e^	Logistic regression		Development model		Mammographic features		Yes		0.58 (0.57-0.60)	
	Percentage density model^b^	Logistic regression		Development model		Mammographic features		Yes		0.54 (0.52-0.56)	
	Deep learning risk score + dense area + percentage density model^f^	Deep neural network		Development model		Mammographic images, the age at image acquisition, exposure, tube current, breast thickness, and compression force		Yes		0.66 (0.64-0.67)	
**Arefan et al [32], 2020**											
	End-to-end convolutional neural network model using GoogLeNet	Convolutional neural network		Development model		Imaging features of the whole-breast region		Yes		0.62 (0.58-0.66)	
	End-to-end convolutional neural network model using GoogLeNet	Convolutional neural network		Development model		Imaging features of the dense breast region only		Yes		0.67 (0.61-0.73)	
	GoogLeNet combining a linear discriminant analysis model	Linear discriminant analysis		Development model		Imaging features of the whole-breast region		Yes		0.64 (0.58-0.70)	
	GoogLeNet combining a linear discriminant analysis model^e,f^	Linear discriminant analysis		Development model		Imaging features of the dense breast region only		Yes		0.72 (0.67-0.76)	
	Area-based percentage breast density model^b^	Logistic regression		Development model		Percentage breast density		Yes		0.54 (0.49-0.59)	
**Tan et al [33], 2013**											
	Support vector machine classification model^e,f^	Support vector machine classification		Validation model		Age, family history, breast density, mean pixel value difference, mean value of short run emphasis; maximum value of short run emphasis, standard deviation of the r-axis cumulative projection histogram, standard deviation of the y-axis cumulative projection histogram, median of the x-axis cumulative projection histogram, mean pixel value, mean value of short run low gray-level emphasis, and median of the x-axis cumulative projection histogram		Yes		0.725 (0.689-0.759)	
**Saha et al [34], 2019**											
	Mean reader scores model^b^	Logistic regression		Development model		—		Yes		0.59 (0.49-0.70)	
	Median reader scores model^b^	Logistic regression		Development model		—		Yes		0.60 (0.51-0.69)	
	Machine learning model 1	Machine learning logistic regression		Development model		Magnetic resonance image background parenchymal enhancement features were based on the fibroglandular tissue mask on the fat saturated sequence		Yes		0.63 (0.52-0.73)	
	Machine learning model 2^e,f^	Machine learning logistic regression		Development model		Magnetic resonance image background parenchymal enhancement features were based on the fibroglandular tissue segmentation using the non–fat-saturated sequence		Yes		0.70 (0.60-0.79)	

^a^AUC: area under the curve.

^b^Traditional risk factor–based optimal breast cancer risk prediction model.

^c^Not available.

^d^BI-RADS: Breast Imaging-Reporting And Data System.

^e^Nonneural network–based optimal breast cancer risk prediction model.

^f^Machine learning–based optimal breast cancer risk prediction model.

^g^BOADICEA: Breast and Ovarian Analysis of Disease Incidence and Carrier Estimation Algorithm.

^h^95% CI not available.

### Study Quality

PROBAST was used to assess the quality of the included studies in terms of both ROB and clinical applicability. All 8 studies demonstrated a low applicability risk; only 1 of the papers had low ROB [7], indicating that most machine learning models have technical pitfalls (Table 3). The other 7 studies that had high ROB were mostly in the domain of analysis, with several reasons as follows: (1) no information was provided on how the continuous/categorical predictors handle or they were handled unreasonably, (2) complexities in the data were not assessed in the final analysis, (3) model calibration was not assessed or lack of standardized evaluation of model calibration, (4) the calculation formulae of the predictors and their weights were not reported in the final model, and (5) insufficient number of participants was used to develop the models. The details are shown in Multimedia Appendix 2. Only 3 neural network–based models were developed by bootstrap and cross-validation to evaluate the discrimination ability of the prediction model, whereas other machine learning models and regression models were developed by using random split or nonrandom split.

**Table 3:** Presentation of the Prediction Model Risk of Bias Assessment Tool results of the included studies.

Study	Risk of bias					Applicability				Overall	
	Participants	Predictors	Outcome	Analysis	Participants		Predictors	Outcome	Risk of bias		Applicability
Yala et al [7], 2021	LR^a^	LR	LR	HR^b^	LR		LR	LR	LR		LR
Ming et al [19], 2020	LR	HR	LR	HR	LR		LR	LR	HR		LR
Portnoi et al [20], 2019	LR	LR	LR	HR	LR		LR	LR	HR		LR
Stark et al [21], 2019	LR	LR	LR	HR	LR		LR	LR	HR		LR
Dembrower et al [31], 2020	LR	LR	LR	HR	LR		LR	LR	HR		LR
Arefan et al [32], 2020	LR	LR	LR	HR	LR		LR	LR	HR		LR
Tan et al [33], 2013	LR	LR	LR	HR	LR		LR	LR	HR		LR
Saha et al [34], 2019	LR	LR	LR	HR	LR		LR	LR	HR		LR

^a^LR: low risk.

^b^HR: high risk.

### Predictive Performance

**Figure 2:**
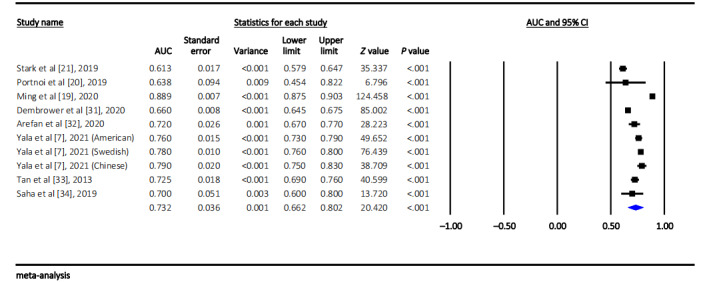
Forest plot of the pooled area under the curve of the machine learning–based optimal breast cancer risk prediction model [7,19-21,31-34]. AUC: area under the curve.

**Figure 3:**
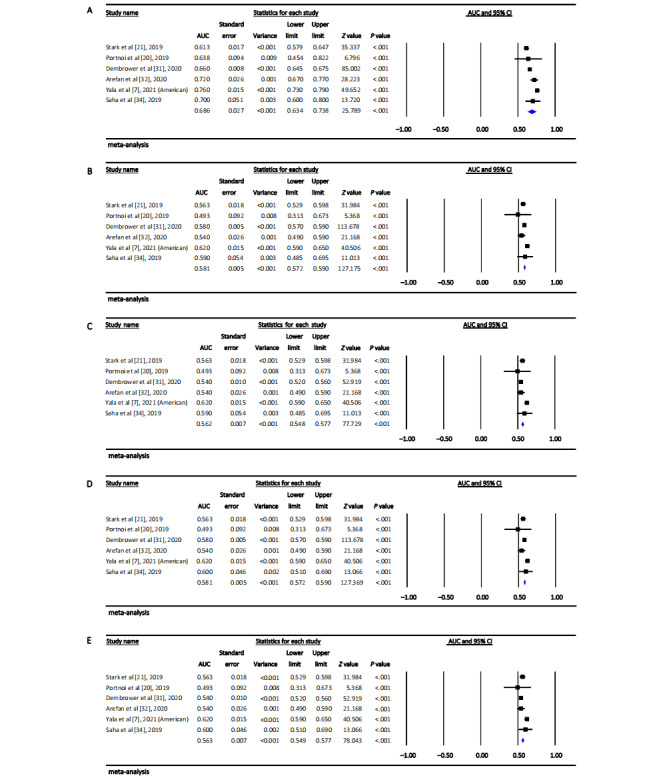
Forest plot of the pooled area under the curve in head-to-head comparisons of (A) machine learning models and (B,C,D,E) traditional risk factor–based models [7,20,21,31,32,34]. AUC: area under the curve.

The pooled AUC of the machine learning–based optimal breast cancer risk prediction model reported in each included study was 0.73 (95% CI 0.66-0.80; approximate 95% PI 0.56-0.96), with a high level of heterogeneity between studies (*Q*=576.07, *I*^2^=98.44%; *P*<.001) (Figure 2). We also performed metaregression, and the results showed that the heterogeneity remains high and essentially unchanged. Sensitivity analysis showed that the pooled AUC and 95% CI were not significantly altered before and after the omission of each data set, with a range of 0.72 (95% CI 0.67-0.76; approximate 95% PI 0.60-0.85) to 0.75 (95% CI 0.68-0.82; approximate 95% PI 0.57-0.98) (Multimedia Appendix 3). The results of head-to-head comparison of the performance difference in both types of models trained by the same data set showed that the pooled AUC of machine learning prediction models (0.69, 95% CI 0.63-0.74; approximate 95% PI 0.57-0.83; Figure 3A) was higher than that of the traditional risk factor–based models, with the range from 0.56 (95% CI 0.55-0.58; approximate 95% PI 0.51-0.62) to 0.58 (95% CI 0.57-0.59; approximate 95% PI 0.51-0.62) (all *P*_heterogeneity_<.001) (Figures 3B-3E).

**Figure 4:**
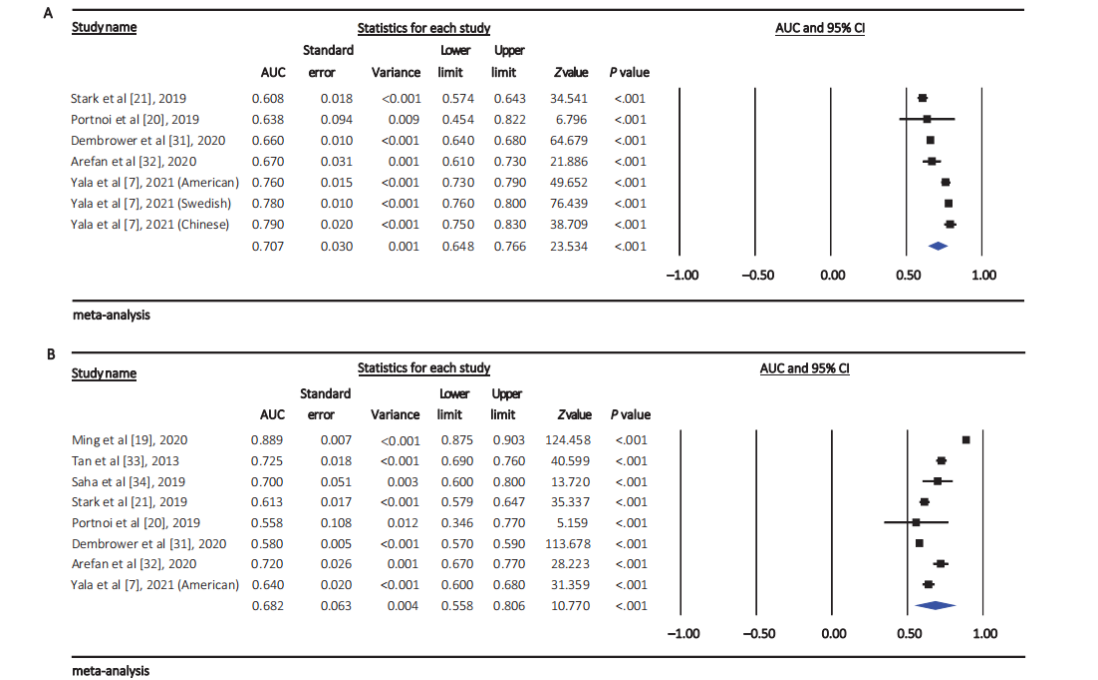
Forest plot of the pooled area under the curve of the (A) neural network–based breast cancer risk prediction model and (B) nonneural network–based optimal risk prediction model [7,20,21,31,32]. AUC: area under the curve.

The pooled AUC of neural network–based breast cancer risk prediction models was 0.71 (95% CI 0.65-0.77; approximate 95% PI 0.57-0.87; *Q*=131.42; *I*^2^=95.43%; *P*<.001) (Figure 4A), which was higher than that of nonneural network–based optimal risk prediction models (0.68, 95% CI 0.56-0.81; approximate 95% PI 0.53-0.81; *Q*=1268.99; *I*^2^=99.45%; *P*<.001) (Figure 4B). When stratified by the presence or absence of incorporation of imaging features, the pooled AUCs in models incorporated with imaging features and those in models not incorporated with imaging features were 0.73 (95% CI 0.67-0.79) and 0.61 (95% CI 0.57-0.64) (*P*_heterogeneity_=.001), respectively (Table 4). Subgroup analysis also showed that the pooled AUC in models not incorporated with genetic risk factors was not significantly lower than that in models incorporated with genetic risk factors (0.71 vs 0.76, respectively; *P*_heterogeneity_=.12) (Table 4). Our results also showed that models predicting short-term (≤5 year) breast cancer risk had a slightly higher pooled AUC than those predicting long-term risk (0.72 vs 0.66, respectively), although the difference was not significant (*P*_heterogeneity_=.10) (Table 4).

The funnel plot indicated that there was no publication bias, with an Egger regression coefficient of –3.85 (*P*=.46) (Multimedia Appendix 4). According to the trim-and-fill method, 2 studies had to be trimmed, and the adjusted pooled AUC was 0.75 (95% CI 0.69-0.82) after trimming (Multimedia Appendix 4).

**Table 4:** Subgroup analysis.

Model, subgroup		Area under the curve (95% CI)	*P*_heterogeneity_ value
**Model with/without imaging features**			.001
	Model incorporated with imaging features	0.73 (0.67-0.79)	
	Model not incorporated with imaging features	0.61 (0.57-0.64)	
**Model with/without** **genetic risk factors**			.12
	Model incorporated with genetic risk factors	0.76 (0.73-0.80)	
	Model not incorporated with genetic risk factors	0.71 (0.65-0.77)	
**Model prediction of risk**			.10
	Model predicting short-term risk	0.72 (0.65-0.78)	
	Model predicting long-term risk	0.66 (0.64-0.67)	

## Discussion

### Principal Findings

In this meta-analysis, 8 studies showed that the pooled AUC of machine learning–based breast cancer risk prediction models was 0.73 (95% CI 0.66-0.80). The results of head-to-head comparison of the performance difference in 2 types of models trained by the same data set showed that machine learning models had a slightly higher advantage than the traditional risk factor–based models in predicting future breast cancer risk. Machine learning approaches have the potential to achieve better accuracy and incorporate different types of information, including traditional risk factors, imaging features, genetic data, and clinical factors. However, of note, the predictive ability of the machine learning models showed substantial heterogeneity among the studies included in this review.

Machine learning represents a data-driven method; it has the ability to learn from past examples and detect hard-to-discern patterns from large and noisy data sets and model nonlinear and more complex relationships by employing a variety of statistical, probabilistic, and optimization techniques [35]. This capability of machine learning algorithms offers a possibility for the investigation and development of risk prediction and diagnostic prediction models in cancer research [36]. It is evident that the use of machine learning methods can improve our understanding of cancer occurrence and progression [35,37]. Thus, developing machine learning–based breast cancer risk prediction models with improved discriminatory power can stratify women into different risk groups, which are useful for guiding the choice for personalized breast cancer screening in order to achieve a good balance in the risk benefit and cost benefit for breast cancer screening.

In our stratified analysis, neural network–based breast cancer risk prediction models incorporating imaging features showed superior performance. This result suggests that the incorporation of imaging inputs in machine learning models can deliver more accurate breast cancer risk prediction. Previous breast cancer risk assessments have already recognized the importance of imaging features in mammography [10,12], but the existing model was based on the underlying pattern that was assessed visually by radiologists, and the whole image was subjectively summarized as a density score on mammography as the model input [38]. It is unlikely that the single value of the density score would be able to take maximum advantage of the imaging features. The other human-specified features may not be able to capture all the risk-relevant information in the image. However, the flexibility of the neural networks might allow the extraction of more information from both finer patterns as well as the overall image characteristics, which can improve the accuracy of the prediction models.

The findings in this study showed that neural network–based models that predicted short-term (≤5 year) breast cancer risk had slightly better discriminatory accuracy than models predicting long-term risk, although confidence intervals overlapped. Improvement of public health literacy and the popularization of healthy lifestyles motivated more opportunities for women in their lifetime to participate in breast cancer prevention and screening and modify their identified modifiable risk factors associated with breast cancer. Unlike many currently known risk factors that do not change and maintain constant risk values, short-term risk factors may change over time. The cumulative effect of these changes may reduce the incidence of breast cancer. Therefore, it is unreasonable to predict the long-term risk of breast cancer by using these risk factors, which may lead to high probability of false-positive recall.

### Model Reliability and Clinical Feasibility

Our study showed several issues regarding machine learning model reliability. The PROBAST analysis indicated that machine learning models have technical pitfalls. First, most machine learning models did not report sufficient statistical analysis information, and only few studies [7,31] provided the details for model reproduction. Second, many machine learning models showed a poor calibration analysis, indicating that the assessment of their utility was problematic, leading to inaccurate evaluation of the future breast cancer risk. Third, only 1 study [7] reported machine learning models that were externally validated in different ethnic populations. Six neural network–based models incorporated many complex imaging features, which may cause clinicians or public physicians to be unable to quickly and conveniently calculate the breast cancer risk by machine learning models manually. This may also be why few studies carry out external validation of the machine learning models. Due to the complexity of the machine learning model algorithms, many studies included many different types of predictors into the model construction, which may lead to an overfitting of the machine learning models [39]. However, only few development studies [7,21,34] reported the details for these predictor selection processes, which may lower the clinical feasibility of the machine learning models.

### Limitations

This review had several limitations. First, most of the included studies [19,31-34] did not provide the expected/observed ratio or other indicators that could evaluate the calibration of the risk prediction model; therefore, this meta-analysis could not comprehensively review the calibration of the machine learning–based breast cancer risk prediction models. Second, substantial heterogeneity was presented in this systematic review, which impeded us from making further rigorous comparisons. The heterogeneity can be partially explained but could not be markedly diminished by different risk predicting times, with or without the incorporation of imaging features and genetic risk factors. The results of meta-analysis can only be interpreted carefully within the context. Third, the pooled results of the machine learning prediction model were analyzed based on most of the included studies that had high ROB [19-21,31-34]. The reason that these studies are rated as high ROB were that complexities in the data were not assessed or the calculation formulas of the predictors and their weights were not reported in the final model. These parameters, the so-called “black boxes,” are almost never presented in the original studies. Moreover, we performed a head-to-head fair comparison of the performance difference between 2 types of models trained by same data set, and the results showed that machine learning models had a slightly higher advantage in predicting future breast cancer risk. Lastly, we mainly focus on the statistical measures of model performance and did not discuss how to meta-analyze the clinical measures of performance such as net benefit. Hence, further research on how to meta-analyze net benefit estimates should be performed.

### Conclusions

In summary, machine learning–based breast cancer risk prediction models had a slightly higher advantage in predicting future breast cancer risk than traditional risk factor–based models in head-to-head comparisons of the performance under the same experimental settings. However, machine learning–based breast cancer risk prediction models had some technical pitfalls, and their clinical feasibility and reliability were unsatisfactory. Future research may be worthwhile to obtain individual participant data to investigate in more detail how the machine learning models perform across different populations and subgroups. We also suggest that they could be considered to be implemented by pooling with breast cancer screening programs and to help developing optimal screening strategies, especially screening intervals.

## Appendix

Multimedia Appendix 1 Search strategy.

Multimedia Appendix 2 Details of risk of bias and the clinical applicability of the included studies.

Multimedia Appendix 3 Sensitivity analysis of the pooled area under the curve of the machine learning–based breast cancer risk prediction models.

Multimedia Appendix 4 Funnel plot of the discrimination of (A) machine learning–based breast cancer risk prediction model and (B) funnel plot adjusted by the trim-and-fill method. AUC: area under the curve.

## Abbreviations

AUC: area under the curve
PI: prediction interval
PRISMA: Preferred Reporting Items for Systematic Reviews and Meta-Analysis
PROBAST: Prediction Model Risk of Bias Assessment Tool
ROB: risk of bias
