# Supplementary material for: An Assessment of the Predictive Performance of Current Machine Learning–Based Breast Cancer Risk Prediction Models: Systematic Review
Source: JMIR Public Health Surveill. 2022 Dec 29;8(12):e35750. doi: 10.2196/35750 (PMC9837707; doi:10.2196/35750)
Supplement: Multimedia Appendix 1 [file publichealth_v8i12e35750_app1.docx]

**Multimedia Appendix 1. Search strategy**

**Text S1 Search strategy**

**Database: PubMed from inception to Present> (Search date: June 9, 2021)**

***Cancer terms:***

1 "breast cancer"[Mesh]

2 (mammary* OR breast cancer* OR breast oncology* OR breast neoplasm* OR breast carcinoma* OR breast tumor* OR breast tumour* OR breast malignant* )[Title/Abstract]

3 1 or 2

***Machine Learning terms:***

4 "Machine learning"[Mesh]

5 "Deep learning"[Mesh]

6 "Neural networks"[Mesh]

7 "Artificial intelligence"[Mesh]

8 (machine learning OR deep learning OR neural networks OR Artificial intelligence)[Title/Abstract]

9 or 4-8

***Risk Prediction terms:***

10 "Risk prediction"[Mesh]

11 "Risk assessment"[Mesh]

12 (risk prediction OR risk assessment) [Title/Abstract]

13 or 10-12

***Study design terms:***

14 "Retrospective Studies"[Mesh]

15 "Cohort Studies"[Mesh]

16 "Longitudinal Studies"[Mesh]

17 "Follow-Up Studies"[Mesh]

18 "Prospective Studies"[Mesh]

19 (cohort or longitudinal or followup or prospective*or retrospective* or database* or population* or follow up)[Title/Abstract]

20 "Registries"[Mesh]

21 (registry or registries) [Title/Abstract]

22 or/14-21

***Final search results: Combining Breast cancer and Machine learning and Risk prediction and Study design:***

23 3 and 9 and 13 and 22

**Text S2 Search strategy**

**Database: EMBASE (Search date: June 9, 2021)**

**Search Strategy:**

***Cancer terms:***

1 'breast cancer'/exp

2 (mammary* or breast cancer* or breast oncology* or breast neoplasm* or breast carcinoma* or breast tumor* or breast tumour* or breast malignant*):ab.ti.

3 1 or 2

***Machine Learning terms:***

4 'Machine learning'/exp

5 'Deep learning'/exp

6 'Neural networks'/exp

7 'Artificial intelligence'/exp

8 (machine learning OR deep learning OR neural networks OR Artificial intelligence):ab.ti.

9 or 4-8

***Risk Prediction terms:***

10 'Risk prediction'/exp

11 'Risk assessment'/exp

12 (risk prediction OR risk assessment):ab.ti.

13 or 10-12

***Study design terms:***

14 'retrospective study'/exp

15 'cohort analysis'/exp

16 'longitudinal study'/exp

17 'follow up'/exp

18 'prospective study'/exp

19 (cohort or longitudinal or followup or prospective*or retrospective* or database* or population* or follow up) :ab.ti.

20 'register'/exp

21 (registry or registries) :ab.ti.

22 or/14-21

***Final search results: Combining Diabetes and Cancer survivor and Study design:***

22 3 and 9 and 13 and 22

**Text S3 Search strategy**

**Database: Cochrane Library from inception to Present> (Search date: December 15, 2020)**

**Search Strategy:**

***Cancer Survivor terms:***

#1 MeSH descriptor: [breast cancer] explode all trees

#2 (breast cancer* or breast oncology* or breast neoplasm* or breast carcinoma* or breast tumor* or breast tumour* or breast malignant*):ti,ab,kw

#3 #1 or #2

***Machine Learning terms:***

#4 MeSH descriptor: [Machine learning] explode all trees

#5 MeSH descriptor: [Deep learning] explode all trees

#6 MeSH descriptor: [Neural networks] explode all trees

#7 MeSH descriptor: [Artificial intelligence] explode all trees

#8 (machine learning OR deep learning OR neural networks OR Artificial intelligence):ti,ab,kw

#9 or/#4-8

***Risk Prediction terms:***

#10 MeSH descriptor: [Risk prediction] explode all trees

#11 MeSH descriptor: [Risk assessment] explode all trees

#12 (risk prediction OR risk assessment):ti,ab,kw

#13 or/#10-12

***Study design terms:***

#14 MeSH descriptor: [Retrospective Studies] explode all trees

#15 MeSH descriptor: [Cohort Studies] explode all trees

#16 MeSH descriptor: [Longitudinal Studies] explode all trees

#17 MeSH descriptor: [Follow-Up Studies] explode all trees

#18 MeSH descriptor: [Prospective Studies] explode all trees

#19 (cohort or longitudinal or followup or prospective*or retrospective* or database* or population* or follow up):ti,ab,kw

#20 MeSH descriptor: [Registries] explode all trees

#21 (registry or registries):ti,ab,kw

#22 or/#14-21

***Final search results: Combining Diabetes and Cancer survivor and Study design:***

#18 #3 and #9 and #13 and #22
