# Supplementary material for: An Assessment of the Predictive Performance of Current Machine Learning–Based Breast Cancer Risk Prediction Models: Systematic Review
Source: JMIR Public Health Surveill. 2022 Dec 29;8(12):e35750. doi: 10.2196/35750 (PMC9837707; doi:10.2196/35750)
Supplement: Multimedia Appendix 2 [file publichealth_v8i12e35750_app2.docx]

Multimedia Appendix 2. Detail of ROB and clinical applicability of included studies.

| Study ID | Participants | | Predictors | | | Outcome | | | | | | Analysis | | | | | | | | |
| --- | --- | --- | --- | --- | --- | --- | --- | --- | --- | --- | --- | --- | --- | --- | --- | --- | --- | --- | --- | --- |
|  | 1.1 | 1.2 | 2.1 | 2.2 | 2.3 | 3.1 | 3.2 | 3.3 | 3.4 | 3.5 | 3.6 | 4.1 | 4.2 | 4.3 | 4.4 | 4.5 | 4.6 | 4.7 | 4.8 | 4.9 |
| Yala et al [7], 2021 | Y^a^ | Y | Y | Y | Y | Y | Y | Y | Y | Y | Y | Y | Y | Y | Y | Y | Y | Y | Y | PY^a^ |
| Ming et al [19], 2020 | Y | Y | Y | Y | N^b^ | Y | Y | Y | Y | Y | Y | Y | NI^c^ | Y | Y | Y | NI | NI | Y | NI |
| Portnoi et al [20], 2019 | Y | Y | Y | Y | Y | Y | Y | Y | Y | Y | Y | Y | NI | N | N | Y | NI | Y | Y | NI |
| Stark et al [21], 2019 | Y | Y | Y | Y | Y | Y | Y | Y | Y | Y | Y | Y | Y | Y | N | Y | NI | NI | Y | NI |
| Dembrower et al [31], 2020 | Y | Y | Y | Y | Y | Y | Y | Y | Y | Y | Y | Y | NI | Y | Y | Y | NI | NI | Y | NI |
| Arefan et al [32], 2020 | Y | Y | Y | Y | Y | Y | Y | Y | Y | Y | Y | N | NI | Y | Y | Y | NI | NI | Y | NI |
| Tan et al [33], 2013 | Y | Y | Y | Y | Y | Y | Y | Y | Y | Y | Y | N | NI | Y | Y | Y | NI | NI | Y | NI |
| Saha et al [34], 2019 | Y | Y | Y | Y | Y | Y | Y | Y | Y | Y | Y | Y | Y | Y | Y | Y | NI | Y | Y | NI |

^a^Y/PY: yes/probably yes.

^b^N/PN: no/probably.

^c^NI: not applicable.
