# Supplementary material for: An Assessment of the Predictive Performance of Current Machine Learning–Based Breast Cancer Risk Prediction Models: Systematic Review
Source: JMIR Public Health Surveill. 2022 Dec 29;8(12):e35750. doi: 10.2196/35750 (PMC9837707; doi:10.2196/35750)
Supplement: Multimedia Appendix 3 [file publichealth_v8i12e35750_app3.docx]

Multimedia Appendix 3. Sensitivity analysis of the pooled AUC of the machine learning-based breast cancer risk prediction models.

| Omitted Study | AUC^a^ | 95%CI^b^ | 95%PI^c^ | *Q* vaule | *I*^2^ | *Tau*^2^ | *P* value |
| --- | --- | --- | --- | --- | --- | --- | --- |
| Ming et al [19], 2020 | 0.715 | 0.668-0.763 | 0.604-0.847 | 147.30 | 94.57 | 0.004 | < .001 |
| Yala et al [7], 2021 (Chinese) | 0.725 | 0.649-0.802 | 0.539-0.975 | 574.77 | 98.61 | 0.013 | < .001 |
| Yala et al [7], 2021 (Swedish) | 0.726 | 0.642-0.809 | 0.528-0.997 | 574.20 | 98.61 | 0.015 | < .001 |
| Yala et al [7], 2021 (American) | 0.728 | 0.650-0.807 | 0.541-0.980 | 575.84 | 98.61 | 0.013 | < .001 |
| Arefan et al [32], 2020 | 0.733 | 0.658-0.809 | 0.551-0.975 | 572.56 | 98.60 | 0.012 | < .001 |
| Tan et al [33], 2013 | 0.733 | 0.656-0.810 | 0.546-0.985 | 570.19 | 98.60 | 0.013 | < .001 |
| Saha et al [34], 2019 | 0.735 | 0.661-0.809 | 0.553-0.976 | 574.33 | 98.61 | 0.012 | < .001 |
| Portnoi et al [20], 2019 | 0.738 | 0.666-0.811 | 0.556-0.979 | 574.18 | 98.61 | 0.012 | < .001 |
| Dembrower et al [31], 2020 | 0.742 | 0.675-0.809 | 0.579-0.950 | 314.33 | 97.46 | 0.009 | < .001 |
| Stark et al [21], 2019 | 0.747 | 0.675-0.818 | 0.570-0.980 | 492.53 | 98.38 | 0.011 | < .001 |

^a^AUC: area under the curve.

^b^CI: confidence interval.

^c^PI: prediction interval.
