# Supplementary figures and images for: An Assessment of the Predictive Performance of Current Machine Learning–Based Breast Cancer Risk Prediction Models: Systematic Review
Source: JMIR Public Health Surveill. 2022 Dec 29;8(12):e35750. doi: 10.2196/35750 (PMC9837707; doi:10.2196/35750)

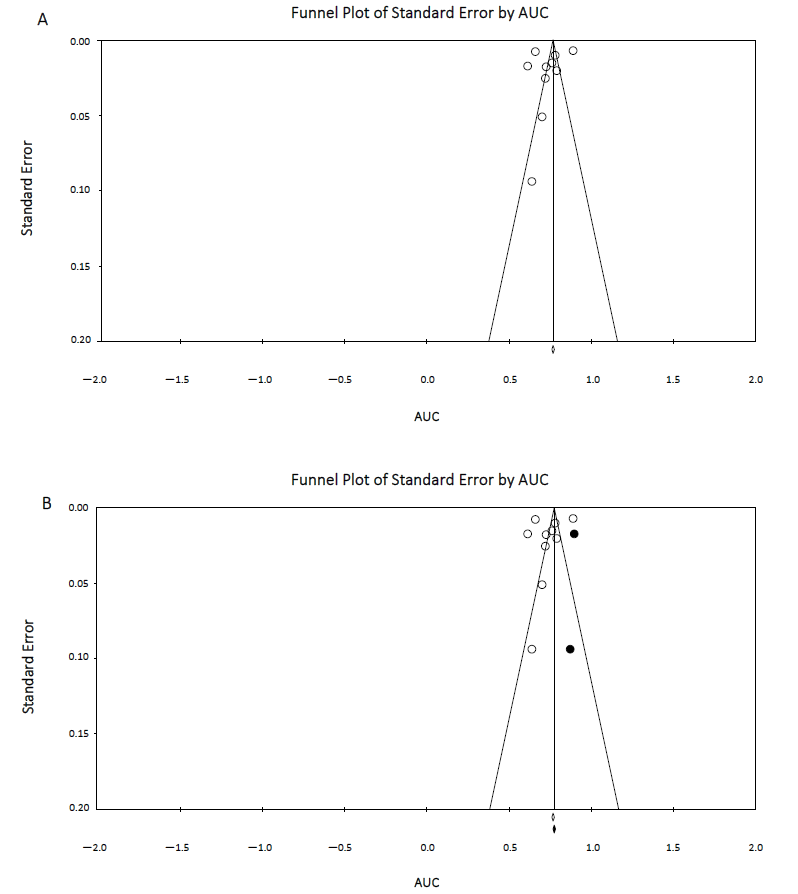

Supplement: Multimedia Appendix 4 [file publichealth_v8i12e35750_app4.png]
